# Mesmeric Infirmary

**Published:** 1845-09

**Authors:** 


*Mesmeric Infirmary.*—It may appear incredible to persons abroad, who are familiar with the educational efforts made in the city of Boston, that an imposition, regarded by the people generally as one really and truly belonging to the catalogue of humbugs, should or could be tolerated here. A hand-bill is circulating, announcing that a Dr. Taylor has opened a *Mesmeric Infirmary*, in Lowell street, in connection with one Tarbox—"who is well known as one of the best clairvoyants to be found in the United States, for the examination of diseases." No doubt exists on that point. The bill further heralds the astounding information that "the services of other clairvoyants for examining by hair, and for traveling clairvoyance, have also been secured. Magnetic, botanic, and all other medicines usually prescribed by clairvoyants, kept constantly on hand." This is an item in the local history of Boston, for 1845!

A first impression would be, that no one possessed of a grain of common sense could be duped by such an unblushing system of deception; but it is altogether probable that it will prove a very money-making device. As far as our own observation extends in regard to these constantly coming and going efforts to profit by the credulity of mankind, strangers from the country are their chief prop and support. It would not be at all surprising to hear that a crowd of persons were seeking advice from this new oracle of mesmerism, and making their pockets as empty as their heads before leaving the premises.—*Boston Med. and Surg. Jour.*

